# Supplementary material for: Recording of intellectual disability in general hospitals in England 2006–2019: Cohort study using linked datasets
Source: PLoS Med. 2023 Mar 20;20(3):e1004117. doi: 10.1371/journal.pmed.1004117 (PMC10069786; doi:10.1371/journal.pmed.1004117)
Supplement: S1 Table — (DOCX) [file pmed.1004117.s002.docx]

**S1 Table** ICD-10 codes for specific disorders almost always associated with intellectual disability, from [1]

| **ICD-10 code** | **Disorder** |
| --- | --- |
| D82.1 | DiGeorge syndrome |
| E00.0 | Congenital iodine-deficiency syndrome, neurological type |
| E00.1 | Congenital iodine-deficiency syndrome, myxedematous type |
| E00.2 | Congenital iodine-deficiency syndrome, mixed type |
| E00.9 | Congenital iodine-deficiency syndrome, unspecified |
| E70.0 | Classical phenylketonuria |
| E77.1 | Defects in glycoprotein degradation |
| E77.8 | Other disorders of glycoprotein metabolism |
| E79.1 | Lesch-Nyhan syndrome |
| E79.8 | Other disorders of purine and pyrimidine metabolism |
| F83 | Mixed specific developmental disorders |
| F84.2 | Rett's syndrome |
| F84.3 | Other childhood disintegrative disorder |
| F84.4 | Overactive disorder associated with mental retardation and stereotyped movements |
| F84.8 | Other pervasive developmental disorders |
| F84.9 | Pervasive developmental disorder, unspecified |
| Q00 | Anencephaly and similar malformations |
| Q00.0 | Anencephaly |
| Q00.1 | Craniorachischisis |
| Q00.2 | Iniencephaly |
| Q04.1 | Arhinencephaly |
| Q04.2 | Holoprosencephaly |
| Q04.3 | Other reduction deformities of brain |
| Q04.8 | Other specified congenital malformations of brain |
| Q85.1 | Tuberous sclerosis |
| Q87.8 | Other specified congenital malformation syndromes, not elsewhere classified |
| Q90 | Down's syndrome |
| Q90.0 | Trisomy 21, nonmosaicism (meiotic nondisjunction) |
| Q90.2 | Trisomy 21, translocation |
| Q90.9 | Down's syndrome, unspecified |
| Q91 | Edwards' syndrome and Patau's syndrome |
| Q91.0 | Trisomy 18, nonmosaicism (meiotic nondisjunction) |
| Q91.1 | Trisomy 18, mosaicism (mitotic nondisjunction) |
| Q91.2 | Trisomy 18, translocation |
| Q91.3 | Trisomy 18, unspecified |
| Q91.4 | Trisomy 13, nonmosaicism (meiotic nondisjunction) |
| Q91.5 | Trisomy 13, mosaicism (mitotic nondisjunction) |
| Q91.6 | Trisomy 13, translocation |
| Q91.7 | Trisomy 13, unspecified |
| Q92 | Other trisomies and partial trisomies of the autosomes, not elsewhere classified |
| Q92.0 | Whole chromosome trisomy, nonmosaicism (meiotic nondisjunction) |
| Q92.1 | Whole chromosome trisomy, mosaicism (mitotic nondisjunction) |
| Q92.2 | Partial trisomy |
| Q92.3 | Minor partial trisomy |
| Q92.7 | Triploidy and polyploidy |
| Q93.3 | Deletion of short arm of chromosome 4 |
| Q93.4 | Deletion of short arm of chromosome 5 |
| Q99.2 | Fragile X chromosome |

**References**

1. Glover, G, Ayub, M. How people with learning disabilities die. Improving Health and Lives: Learning Disabilities Observatory. Department of Health; 2010.
