# Supplementary material for: Recording of intellectual disability in general hospitals in England 2006–2019: Cohort study using linked datasets
Source: PLoS Med. 2023 Mar 20;20(3):e1004117. doi: 10.1371/journal.pmed.1004117 (PMC10069786; doi:10.1371/journal.pmed.1004117)
Supplement: S2 Table — (DOCX) [file pmed.1004117.s003.docx]

**S2 Table** Time trends in recording intellectual disability in adults admitted to English general hospitals, 2005-2019

A) number with intellectual disability recorded with F70-79 (intellectual disability) and F70-79 *or* F81 (intellectual disability or disorder of scholastic skills not otherwise specified) (first emergency admission during cohort)

|  | **2005** | **2006** | **2007** | **2008** | **2009** | **2010** | **2011** | **2012** | **2013** | **2014** | **2015** | **2016** | **2017** | **2018** | **2019** | **Total** |
| --- | --- | --- | --- | --- | --- | --- | --- | --- | --- | --- | --- | --- | --- | --- | --- | --- |
| **F70-F79** | | | | | | | | | | | | | | | | |
| No (FN) | 375 | 274 | 205 | 203 | 222 | 139 | 141 | 141 | 111 | 116 | 82 | 78 | 79 | 64 | 23 | 2253 |
| Yes (TP) | 7 | 1 | 9 | 3 | 0 | 3 | 1 | 0 | 0 | 1 | 3 | 1 | 7 | 3 | 1 | 40 |
| Total | 382 | 275 | 214 | 206 | 222 | 142 | 142 | 141 | 111 | 117 | 85 | 79 | 86 | 67 | 24 | 2293 |
| **F70-79 or F81** | | | | | | | | | | | | | | | | |
| No (FN) | 315 | 234 | 167 | 161 | 179 | 99 | 91 | 84 | 77 | 74 | 49 | 42 | 51 | 35 | 9 | 1667 |
| Yes (TP) | 67 | 41 | 47 | 45 | 43 | 43 | 51 | 57 | 34 | 43 | 36 | 37 | 35 | 32 | 15 | 626 |
| Total | 382 | 275 | 214 | 206 | 222 | 142 | 142 | 141 | 111 | 117 | 85 | 79 | 86 | 67 | 24 | 2293 |

FN, false negative; TP, true positive

B) proportion with intellectual disability recorded with F70-79 (intellectual disability) and F70-79 *or* F81 (intellectual disability or disorder of scholastic skills not otherwise specified) (first emergency admission during cohort)

|  | **2005** | **2006** | **2007** | **2008** | **2009** | **2010** | **2011** | **2012** | **2013** | **2014** | **2015** | **2016** | **2017** | **2018** | **2019** |
| --- | --- | --- | --- | --- | --- | --- | --- | --- | --- | --- | --- | --- | --- | --- | --- |
| **F70-F79** | | | | | | | | | | | | | | | |
| Proportion recorded (95%CI) | 1.8 (0.7, 3.7) | 0.4 (0.0, 2.0) | 4.2 (1.9, 7.8) | 1.5 (0.3, 4.2) | 0.0 (0.0, 1.7) | 2.1 (0.4, 6.1) | 0.7 (0.0, 3.9) | 0.0 (0.0, 2.6) | 0.0 (0.0, 3.3) | 0.9 (0.0, 4.7) | 3.5 (0.7, 10.0) | 1.3 (0.0, 6.9) | 8.1 (3.3, 16.1) | 4.5 (0.9, 12.5) | 4.2 (0.1, 21.1) |
| **F70-79 or F81** | | | | | | | | | | | | | | | |
| Proportion recorded (95%CI) | 17.5 (13.8, 21.7) | 14.9 (10.9, 19.7) | 22.0 (16.6, 28.1) | 21.8 (16.4, 28.1) | 19.4 (14.4, 25.2) | 30.3 (22.9, 38.6) | 35.9 (28.0, 44.4) | 40.4 (32.3, 49.0) | 30.6 (22.2, 40.1) | 36.8 (28.0, 46.2) | 42.4 (31.7, 53.6) | 46.8 (35.5, 58.4) | 40.7 (30.2, 51.8) | 36.8 (26.7, 47.8) | 62.5 (40.6, 81.2) |

CI, confidence interval
