# Supplementary material for: Recording of intellectual disability in general hospitals in England 2006–2019: Cohort study using linked datasets
Source: PLoS Med. 2023 Mar 20;20(3):e1004117. doi: 10.1371/journal.pmed.1004117 (PMC10069786; doi:10.1371/journal.pmed.1004117)
Supplement: S3 Table — (DOCX) [file pmed.1004117.s004.docx]

**S3 Table** Odds of intellectual disability being unrecorded in the general hospital record of adults with intellectual disability attending hospital (with multiple imputation for missing data)

|  |  | **Adjusted analysis*** | |
| --- | --- | --- | --- |
|  |  | **Odds Ratio (95%CI)** | ***p*-value** |
| **Age** | (OR per 10 years older age) | **1.12 (1.05, 1.20)** | **0.001** |
| **Sex** | Female (reference) | 1 | - |
|  | Male | 1.06 (0.85, 1.31) | 0.62 |
| **Degree of intellectual disability**** |  | **0.61 (0.51, 0.73)** | **<0.001** |
| **Ethnicity** | White (reference) | 1 | - |
|  | Asian | 1.26 (0.73, 2.18) | 0.41 |
|  | Black | 1.90 (0.68, 1.20) | 0.48 |
|  | Mixed | 1.09 (0.58, 2.06) | 0.78 |
|  | Other | 0.69 (0.36, 1.32) | 0.26 |
| **Marital status** | Unmarried (reference) | 1 | - |
|  | Married | **2.09 (1.09, 4.00)** | **0.03** |
| **Deprivation index** | (OR per decile higher deprivation) | 0.97 (0.87, 1.07) | 0.53 |

*Adjustment for all variables in the table and for number of general hospital admissions during study period

**In the imputed analysis degree of intellectual disability was considered a continuous variable
