# Supplementary material for: Recording of intellectual disability in general hospitals in England 2006–2019: Cohort study using linked datasets
Source: PLoS Med. 2023 Mar 20;20(3):e1004117. doi: 10.1371/journal.pmed.1004117 (PMC10069786; doi:10.1371/journal.pmed.1004117)
Supplement: S4 Table — (DOCX) [file pmed.1004117.s005.docx]

**S4 Table** Odds of intellectual disability being unrecorded in the general hospital record of adults with intellectual disability attending hospital (using a random effects model)

|  | | **Univariate analysis** | | **Adjusted analysis*** | |
| --- | --- | --- | --- | --- | --- |
|  |  | **Odds Ratio (95%CI)** | ***p*-value** | **Odds Ratio (95%CI)** | ***p*-value** |
| **Age** | OR per 10 years older age | **0.82 (0.79-0.86)** | **<0.001** | **0.90 (0.84-0.95)** | **0.001** |
| **Sex** | Female (reference) | 1 | - | 1 | - |
|  | Male | 0.98 (0.82-1.16) | 0.78 | 1.12 (0.92-1.36) | 0.26 |
| **Degree of intellectual disability** | Mild (reference) | 1 | - | 1 | - |
|  | Moderate | 0.94 (0.75-1.19) | 0.59 | 1.01 (0.79-1.29) | 0.95 |
|  | Severe | **0.32 (0.24-0.44)** | **<0.001** | **0.32 (0.23-0.46)** | **<0.001** |
|  | Profound | **0.30 (0.10-0.90)** | **0.03** | **0.26 (0.07-0.92)** | **0.04** |
| **Ethnicity** | White (reference) | 1 | - | 1 | - |
|  | Asian | **0.63 (0.41-0.96)** | **0.03** | **0.61 (0.39-0.96)** | **0.03** |
|  | Black | 1.15 (0.92-1.43) | 0.22 | 1.08 (0.84-1.37) | 0.55 |
|  | Mixed | 1.32 (0.79-2.21) | 0.30 | 0.85 (0.48-1.51) | 0.58 |
|  | Other | 1.25 (0.69-2.26) | 0.45 | 1.00 (0.54-1.87) | 0.99 |
| **Marital status** | Unmarried (reference) | 1 | - | 1 | - |
|  | Married | **3.11 (2.02-4.78)** | **<0.001** | **3.36 (2.10-5.39)** | **<0.001** |
| **Deprivation index** | OR per decile higher deprivation | **1.24 (1.15-1.33)** | **<0.001** | **1.17 (1.07-1.27)** | **<0.001** |
| **Admission type** | Emergency | 1 | - | 1 | - |
|  | Elective | **1.91 (1.74,2.09)** | **<0.001** | **1.97 (1.77, 2.18)** | **<0.001** |

*Adjustment for all variables in the table and for number of general hospital admissions during study period
