# Supplementary material for: Recording of intellectual disability in general hospitals in England 2006–2019: Cohort study using linked datasets
Source: PLoS Med. 2023 Mar 20;20(3):e1004117. doi: 10.1371/journal.pmed.1004117 (PMC10069786; doi:10.1371/journal.pmed.1004117)
Supplement: S5 Table — (DOCX) [file pmed.1004117.s006.docx]

**S5 Table** Odds of intellectual disability being unrecorded in the general hospital record of adults with intellectual disability attending hospital (with multiple imputation for missing data)

|  |  | **Adjusted analysis*** | |
| --- | --- | --- | --- |
|  |  | **Odds Ratio (95%CI)** | ***p*-value** |
| **Age** | OR per 10 years older age | **0.90 (0.85-0.95)** | **<0.001** |
| **Sex** | Female (reference) | 1 | - |
|  | Male | 1.05 (0.88-1.25) | 0.61 |
| **Degree of intellectual disability** | Mild (reference) | 1 | - |
|  | Moderate | 0.89 (0.71-1.13) | 0.34 |
|  | Severe | **0.35 (0.27-0.47)** | **<0.001** |
|  | Profound | **0.31 (0.11-0.89)** | **0.03** |
| **Ethnicity** | White (reference) | 1 | - |
|  | Asian | 0.69 (0.45-1.06) | 0.09 |
|  | Black | 1.06 (0.85-1.32) | 0.60 |
|  | Mixed | 1.05 (0.62-1.77) | 0.86 |
|  | Other | 1.10 (0.61-1.97) | 0.77 |
| **Marital status** | Unmarried (reference) | 1 | - |
|  | Married | **2.60 (1.72-3.90)** | **<0.001** |
| **Deprivation index** | OR per decile higher deprivation | **1.19 (1.11-1.29)** | **<0.001** |
| **Admission type** | Emergency | 1 | - |
|  | Elective | **1.93 (1.76, 2.12)** | **<0.001** |

*Adjustment for all variables in the table
