# Supplementary material for: Recording of intellectual disability in general hospitals in England 2006–2019: Cohort study using linked datasets
Source: PLoS Med. 2023 Mar 20;20(3):e1004117. doi: 10.1371/journal.pmed.1004117 (PMC10069786; doi:10.1371/journal.pmed.1004117)
Supplement: S1 Text — (DOCX) [file pmed.1004117.s007.docx]

**S1 Text** Study protocol

**Sensitivity of intellectual disability recording in English general hospitals and association with readmission**

## **Background**

People with intellectual disability have worse physical health and shorter life expectancy compared to those without [1]. General hospital admission rates are around 3 times higher [2] with admissions often being long and complex where the person can experience adverse outcomes [3] and high re-admission rates [4]. This has been partly attributed to poor quality care [5] and recognising people with intellectual disability in general hospitals is seen as one potential remedy to this.

The ability to provide the most appropriate care in hospital for people with intellectual disability, and make reasonable adjustments to care and treatments to account for the cognitive impairment and potential capacity impairment, partly relies on recognition and accurate formal recording of the intellectual disability. However, we currently have little information on how well intellectual disability is recognised in in-patient settings. One study has compared healthcare commissioners’ recording of UK general hospital admissions with expected admission rates and suggested that rates were 5% lower than expected [6], possibly indicating under-recording but these data are based upon ecological data which is at risk of bias.

In this project, we propose to use routinely collected clinical data from the South London and Maudsley NHS Trust ‘CRIS’ database to identify adults with intellectual disability or autism and then use their linked NHS general hospital records to examine whether the condition was correctly recorded during hospital admissions. As in our previous work [7], we will also consider whether recording accuracy is changing over time and which clinical and demographic factors are associated with diagnostic recording accuracy. Finally, we will consider whether accurate diagnostic recording affects clinical outcomes; we will examine subsequent re-admission rate and consider whether we can examine other potential outcomes i.e. length of stay, mortality, etc.

### **Aims**

1. Determine the sensitivity of diagnosis of intellectual disability or autism in Hospital Episode Statistics for patients with these diagnoses in CRIS
2. Analyse time-trends since 2007
3. Investigate sociodemographic and clinical predictors of accurate diagnostic recording, e.g. age, gender, ethnicity, socioeconomic deprivation, and mental health symptom severity
4. Examine association of diagnostic recording accuracy with subsequent readmission rate

## **Method**

Cohort study using two linked electronic health record databases. We will use the South London and Maudsley National Health Service (NHS) Foundation Trust (SLaM) ‘Clinical Record Interactive Search’ (CRIS) data extraction tool to identify people with intellectual disability or autism, linked to NHS Digitals’ Hospital Episode Statistics to obtain information on hospital diagnoses.

### **Study participants**

We will include study participants who:

1) had clinical contact with SLaM mental health services between 1st January 2006 and 31^st^ March 2017 while aged 18 or over

2) were diagnosed at any time with intellectual disability (ICD-10 code F70-79), or autism (F84) in structured EHR diagnostic fields or unstructured text (using natural language processing (NLP) algorithms developed on General Architecture for Text Engineering (GATE) software

3) were admitted to an English NHS general (non-psychiatric) hospital after the first diagnosis of intellectual disability in SLaM

### **Outcomes**

We will obtain outcome data from Hospital Episode Statistics (HES) [8] records of clinical diagnoses from admissions to any hospital within England until 31^st^ March 2017. The index date will be the first diagnosis of intellectual disability or autism within SLaM and we will extract dates of admission and discharge, admission method (emergency or elective), and up to 20 primary and secondary recorded diagnoses for all admissions of included patients to general (i.e. non-psychiatric) hospitals. We will use data from emergency admissions only as many elective admissions are brief admissions for recurrent treatments such as wound dressing, renal dialysis during which full diagnostic assessment may not need to be undertaken.

### **Covariates**

Derived from SLaM records closest to time of first general hospital admission:

- Age
- Sex
- Ethnicity (White, Mixed, Asian or British Asian, Black or Black British, and other)
- Neighbourhood-level socioeconomic deprivation from the Index of Multiple Deprivation (IMD)
- Health of the Nation Outcome Scale (HoNOS) to rate clinical presentation. We will combine the subscales reflecting mental health symptoms (agitation, self-injury, drug/alcohol use, cognitive impairment, delusions/hallucinations and depressed mood) into a subscale indicating 0 symptoms, 1 current mental health symptom, 2 current mental health symptoms , and 3+ current mental health symptoms and use the physical illness and activity of daily living (ADL) impairment.

### **Analysis**

We will first describe characteristics of sample. We will describe the primary diagnostic codes for hospital admissions to identify reasons for admission and the healthcare provider codes to identify the range of hospital trusts in which patients were admitted.

#### Determine the sensitivity of diagnosis of intellectual disability and autism in Hospital Episode Statistics for patients with these diagnoses in CRIS

We will calculate sensitivity of general hospital records (i.e. proportion of people with intellectual disability and autism SLaM diagnosis who have these diagnoses recorded when admitted to general hospital) for each individual emergency hospital admission ‘admission-level’, and for each person’s whole hospital records ‘person-level’.

#### Analyse time-trends since 2007

We will calculate sensitivity for each participant’s first emergency hospital admission following intellectual disability/autism diagnosis stratified by year of admission and use chi-squared test for trend to examine changes over time.

#### Investigate predictors of accurate diagnostic recording, e.g. age, gender, ethnicity, socioeconomic deprivation, and mental health symptom severity

We will examine the association of sociodemographic and clinical characteristics with unrecorded diagnosis using multivariable logistic regression; included variables will be age, sex, ethnicity, IMD, HoNOS clinical symptoms (number of mental illness symptoms, physical illness, ADL impairment) and number of hospital admissions.

Sensitivity analyses 1) without HoNOS variables as these data have high missingness and 2) using multiple imputation with chained equations to replace missing values.

#### Examine association of diagnostic recording accuracy with subsequent readmission

We will examine the association of whether diagnosis was recorded vs unrecorded during each emergency admission and subsequent time to readmission. This analysis will use multi-level survival analysis with a random effect for each patient, as clustering is likely at patient level. Adjusted for age, sex, ethnicity, neighbourhood-level socioeconomic deprivation and clinical symptoms.

**References**

1. University of Bristol. Learning Disabilities Mortality Review Programme Annual Report 2018. University of Bristol; 2019.
2. Hosking F, Carey I, DeWilde S, Harris T, Beighton C, Cook D. Preventable emergency hospital admissions among adults with intellectual disability in England. Ann Fam Med. 2017;15:462-470.
3. Heslop P, Blair PS, Fleming P, Hoghton M, Marriott A, Russ L. The Confidential Inquiry into premature deaths of people with intellectual disabilities in the UK: a population-based study. Lancet. 2014;383:889–895.
4. Kelly C, Thomson K, Wagner A, Waters J, Thompson A, Jones S, et al. Investigating the widely held belief that men and women with learning disabilities receive poor quality healthcare when admitted to hospital: a single‐site study of 30‐day readmission rates. J Intellect Disabil Res. 2015;59**:**835-844.
5. Mencap. Death by Indifference. London: Mencap; 2007.
6. Glover G, Fox S, Hatton C. General hospital care for people with intellectual disabilities. Tizard Learn Disabil Rev. 2016;21(1):43-49.
7. Sommerlad A, Perera G, Singh-Manoux A, Lewis G, Stewart R, Livingston G. Accuracy of general hospital dementia diagnoses in England: sensitivity, specificity, and predictors of diagnostic accuracy 2008-2016. Alzheimers Dement. 2018;14:933-943.
8. Health and Social Care Information Centre. HES Data Dictionary: Admitted Patient Care. NHS Digital; 2017.
