# Supplementary material for: Recording of intellectual disability in general hospitals in England 2006–2019: Cohort study using linked datasets
Source: PLoS Med. 2023 Mar 20;20(3):e1004117. doi: 10.1371/journal.pmed.1004117 (PMC10069786; doi:10.1371/journal.pmed.1004117)
Supplement: Attachment — Submitted filename: Recording of intellectual disability - author response.docx [file pmed.1004117.s008.docx]

**Recording of intellectual disability in general hospitals in England 2006-2019: cohort study using linked datasets**

**Author response to reviewer comments**

We thank the editor and reviewers for their helpful comments. Comments in black text are followed by author response in red text. We have included additions to the text and given the page and line numbers corresponding to the clean (unmarked) version of the manuscript.

**Requests from the editors:**

Please include the setting (England) in your title.

We have amended the title:

“Recording of intellectual disability in general hospitals in England 2006 and 2019: cohort study using linked datasets”

Abstract Methods and Findings:

* Please include the setting (England).

This has now been added to the abstract (p.2, l.25):

“Retrospective cohort study using two linked datasets of routinely collected clinical data in England.”

* Please include the important covariates that are adjusted for in the analyses.

We have added the requested information (p.2, l.34-37):

“In analyses adjusted for age, sex, ethnicity, and socio-economic deprivation, having a mild intellectual disability and being married were associated with increased odds of the intellectual disability being unrecorded in hospital records.”

At this stage, we ask that you include a short, non-technical Author Summary of your research to make findings accessible to a wide audience that includes both scientists and non-scientists. The Author Summary should immediately follow the Abstract in your revised manuscript. This text is subject to editorial change and should be distinct from the scientific abstract. Please see our author guidelines for more information: <https://eur03.safelinks.protection.outlook.com/?url=https%3A%2F%2Fjournals.plos.org%2Fplosmedicine%2Fs%2Frevising-your-manuscript%23loc-author-summary&amp;data=05%7C01%7Crory.sheehan%40kcl.ac.uk%7C4c6300bc192c4668260008dad21e575b%7C8370cf1416f34c16b83c724071654356%7C0%7C0%7C638053325002381778%7CUnknown%7CTWFpbGZsb3d8eyJWIjoiMC4wLjAwMDAiLCJQIjoiV2luMzIiLCJBTiI6Ik1haWwiLCJXVCI6Mn0%3D%7C3000%7C%7C%7C&amp;sdata=2%2FXaLka5jk1D1LD1wTGWCeUrfTnMV1oABFU8ljB2n8k%3D&amp;reserved=0>

We have included an author summary (p.4-5, l.45-72):

**Why was this study done?**

• People with intellectual disability have higher health needs and are more likely to be	admitted to hospital than people without intellectual disability.

• Existing evidence shows that hospital care for people with intellectual disability needs to	be improved if the worse health outcomes and health inequalities experienced by this group	are to be overcome.

• Intellectual disability needs to be recognised in healthcare settings in order that individual	reasonable adjustments to care can be made.

**What did the researchers do and find?**

• We joined two databases covering from 2006 to 2019 to investigate how often intellectual	disability is recorded when people with the condition are admitted to general hospitals in	England.

• We included 2,477 people with intellectual disability who together had 27,314 admissions	to hospital.

• Intellectual disability was accurately recorded in only 2.9% of all admissions and was more	likely to be unrecorded in people with less severe intellectual disability and those who were	married.

**What do these findings mean?**

• Recognition and recording of intellectual disability in general hospitals in England needs to	be improved and must then be followed by processes and procedures that impact the	quality and effectiveness of care that people receive.

• Using English Hospital Episode Statistics (HES) data alone to identify people with	intellectual disability accessing general hospital care is insufficient and data linkage with	other sources is necessary to obtain a more accurate picture for research and service	planning purposes.

Please ensure that the study is reported according to the RECORD guideline, and include the completed RECORD checklist as Supporting Information. Please add the following statement, or similar, to the Methods: "This study is reported as per the REporting of studies Conducted using Observational Routinely-collected Data (RECORD) guideline (S1 Checklist)."

The RECORD guideline can be found here: <https://eur03.safelinks.protection.outlook.com/?url=http%3A%2F%2Fwww.equator-network.org%2Freporting-guidelines%2FRECORD%2F&amp;data=05%7C01%7Crory.sheehan%40kcl.ac.uk%7C4c6300bc192c4668260008dad21e575b%7C8370cf1416f34c16b83c724071654356%7C0%7C0%7C638053325002381778%7CUnknown%7CTWFpbGZsb3d8eyJWIjoiMC4wLjAwMDAiLCJQIjoiV2luMzIiLCJBTiI6Ik1haWwiLCJXVCI6Mn0%3D%7C3000%7C%7C%7C&amp;sdata=4D%2F2R6mWWD6sZRNkOysl%2BB8oNK2EMCpv9m0gHtQOncI%3D&amp;reserved=0>

When completing the checklist, please use section and paragraph numbers, rather than page numbers.

We have included the RECORD checklist as Supporting Information (S1 checklist).

Please define the abbreviations in Table 1 in the legend.

We have defined SD, standard deviation, and IQR, inter-quartile range (p.14, l.268).

Line 242: Please do not report P=0.0001; report as P < 0.001.

This has been amended in table 3 (p.17).

Please indicate in the figure caption the meaning of the bars in Figure 1.

We have added the meaning (p.16, l.308):

“Bars indicate 95% confidence intervals"

Please define F70-79 and F81.9 in the legend of Figure 1.

We have defined these codes (p.16, l.307-308):

“F70-F79, ICD-10 codes for intellectual disability”

“F81.9, ICD-10 code for developmental disorder of scholastic skills, unspecified”

Please clarify what is denoted by bold typeface in Table 3; if statistical significance, should univariate age also be in bold?

Thank you. Bold does indicate statistical significance and we have corrected the table.

Please use et al. only after listing the first six authors in your References. See our website for other reference guidelines <https://eur03.safelinks.protection.outlook.com/?url=https%3A%2F%2Fjournals.plos.org%2Fplosmedicine%2Fs%2Fsubmission-guidelines%23loc-references&amp;data=05%7C01%7Crory.sheehan%40kcl.ac.uk%7C4c6300bc192c4668260008dad21e575b%7C8370cf1416f34c16b83c724071654356%7C0%7C0%7C638053325002537969%7CUnknown%7CTWFpbGZsb3d8eyJWIjoiMC4wLjAwMDAiLCJQIjoiV2luMzIiLCJBTiI6Ik1haWwiLCJXVCI6Mn0%3D%7C3000%7C%7C%7C&amp;sdata=WpWFz4LnAhUknmtcoXZaLzbpuZzS921NWk3KEAjWgws%3D&amp;reserved=0>

The references have been amended to house style.

**Comments from the reviewers:**

**Reviewer #1:**

This is a well-written paper about the recording of intellectual disabilities in hospital records from 2006 - 2019. This is a topic of importance, but I suggest that a more nuanced consideration of the issues is required before the paper is ready for publication. In particular, I suggest that the authors need to consider the following:

We thank the reviewer for their comments.

1. A definition of hospital admission is required, including how this might relate to hospital attendance.

The Hospital Episode Statistics (HES) dataset includes admissions to hospital, attendances to the emergency department, and out-patient appointments. In this study we included records indicative of an admission to hospital, including elective and emergency in-patient admissions. Visits to the emergency department not resulting in admission and out-patient appointments were not included; investigating recording of intellectual disability during these contacts could be explored in future studies but was not relevant to our central aims.

We have made this clearer in the methods of the manuscript (p.9, l.167-172):

“These individuals’ patient records were linked with HES data over the same period using approved secure processes via the SLaM Clinical Data Linkage Service [28], to identify all general hospital in-patient admissions during the study period and diagnoses recorded within the general hospital setting during each admission. We did not measure attendances to the out-patient department or visits to the emergency department which did not result in an admission.”

2. A justification for why a deprivation index score has been included is needed. Many people with intellectual disabilities live in residential or supported living settings that bear no resemblance to socio-economic status of themselves or their families.

The Index of Multiple Deprivation (IMD) is a standard variable used to approximate an individual’s level of deprivation. We agree with the reviewer that area-based measures of deprivation may not be a good indicator of individual-level deprivation in people with intellectual disability and those living in care homes. However, there are some elements to the deprivation index that are related to the quality of the local environment that are likely to be relevant to all individuals living in that area, and we currently lack any better, readily-available measure of socio-economic status in this group.

We have added to the discussion to highlight that IMD was found not to be associated with the outcome of interest and this may be because of the limitations of IMD as a measure (p.21, l.424-428):

“Other factors investigated were not associated with recording in hospital records; the lack of a consistent association with socio-economic status may reflect the IMD not being a sufficiently robust measure of deprivation in people with intellectual disability, as many live in group settings which do not reflect the socio-economic status of themselves of their families [48].”

3. A justification for the dates for inclusion in the study. This seems to me, to be a convenience sample, but the start date for inclusion, 2006, predates the requirements of the Equality Act 2010, which demands the provision of reasonable adjustments for disabled people. As such, including data before this legislation came into force, and making assumptions that it was already in force, creates a tension in the paper.

The data presented span the time that the dataset has been available (the CRIS data are available from 2006, p8, l.139), ending shortly before the onset of the COVID-19 pandemic, as care delivered after this time was disrupted to a very great extent. We have clarified this in the methods section (p.9, l.161-165):

“We retrieved records of all adults (≥ 18 years) in the SLaM BRC case register with a diagnosis of intellectual disability who had received care from SLaM NHS Trust between 2006 and 2019; this timeframe was selected to maximise the data available for analysis and in order that longitudinal trends could be investigated, finishing prior to the COVID-19 pandemic which greatly impacted in-patient healthcare.”

We consider it is important to include maximum available data so that longitudinal trends can be investigated. Although our study start date pre-dates the Equality Act (2010), the precursor legislation to the Equality Act, the Disability Discrimination Act (1995), did place a responsibility on providers of services to take reasonable steps to ensure that the needs of people with a disability were considered. The health inequalities and poor hospital experiences of people with intellectual disability have been recognised for many years (e.g. Treat Me Right!, Mencap, 2004) and systems responses, including the need to accurately record intellectual disability in those attending hospital, had been suggested soon after (e.g. Healthcare for All, Department of Health, 2008). We have added discussion of the potential impact of the Equality Act and other legislation on our findings that recording changed over time (described in detail below in response to point 5.)

4. More discussion about ICD10 coding is required, and the justification for only including the codes used. Glover, 2006 (file:///C:/Users/saph/Downloads/Howpeoplewithlearningdisabilitiesdie%20(1).pdf) includes a list in Appendix 1 of conditions always associated with intellectual disabilities, with the ICD10 codes. It would be instructive to understand the proportion of admissions with these codes but without F70-79.

We thank the reviewer for raising this point and accept that using only ICD-10 codes F70-F79 will exclude people with specific causes of intellectual disability that are coded in other ICD-10 chapters. We have therefore used the list of conditions suggested by the reviewer to expand the list of recoded diagnoses in HES data that we capture. Using this expanded list of codes means that an additional 793 admissions of the 27,314 in total would be recorded as having intellectual disability, giving an overall sensitivity of recording of 30.6% (increased from 27.7%). Whilst inclusion of specific codes does increase recording by a small amount, the overall conclusions are not changed, with the majority of admissions still not recorded when each admission is considered individually.

We have added this to the methods and results sections (p.11, l.219-221 and p.14-15, l.286-289):

“For each of these we investigated intellectual disability recording in the HES data using:

…

iii) ICD-10 intellectual disability codes (F70-F79), the F81.9 code, and 48 individual ICD-10 codes for specific disorders almost always associated with intellectual disability from a code list developed previously [31]”

“Adding a list of codes for specific disorders associated with intellectual disability resulted in a small increase in the proportion of admissions in which intellectual disability was recorded (admission-level sensitivity = 30.6% (30.1, 31.2); patient-level sensitivity = 69.2% (67.4, 71.1).”

|  | Sensitivity (95% CI) |  |  |
| --- | --- | --- | --- |
| ICD-10 descriptor and codes | Intellectual disability (F70-F79) | Intellectual disability (F70-F79)  Developmental disorder of scholastic skills, unspecified (F81.9) | Intellectual disability (F70-F79)  Developmental disorder of scholastic skills, unspecified (F81.9)  Disorders associated with intellectual disability (*) |
|  |  |  |  |
| For each admission (admission sensitivity) | 2.9 (2.7, 3.1) | 27.7 (27.2, 28.3) | 30.6 (30.1, 31.2) |
| For each patient (patient-level sensitivity) | 18.0 (16.5, 19.5) | 66.3 (64.4, 68.1) | 69.2 (67.4, 71.1) |
| For each admission (emergency admissions only) | 2.0 (1.8, 2.2) | 32.7 (32.0, 33.4) | 36.0 (35.2, 36.7) |

5. The authors acknowledge that the rate of recording rose over time, being very different in 2019 from 2006. More recognition and discussion about this is needed, including suggestions as to the impact of the Equality Act, guidance for healthcare professionals (see for example, guidance for GPs and nursing staff), the introduction of learning disability nurses, findings from inquiries and reviews indicating the need for such recording over the years (e.g. CIPOLD study). A timeline, for example, illustrating the potential impact of such initiatives against the rise in reporting might be helpful. In addition, it might be helpful to present the findings from the most recent 2-3 years separately, so as to present a more contemporary picture of the situation.

We thank the reviewer for these suggestions. We have added to the discussion to include some of the salient legislative and practice changes that occurred over the study period which might account for the improvements in recording. We have not included a timeline as a figure but have indicated in the text the year in which events occurred. We highlight the level of recording in the final year in the discussion (p.20, l.389-412):

“Our data indicate that recording of intellectual disability has improved over time, if the related (but technically incorrect) code of learning difficulty (ICD-10 F81.9) is included. For example, intellectual disability was recorded in over half of new emergency admissions occurring in 2019. The cumulative effect of legislative changes, growing research evidence, and clinical initiatives over the past 15 years may have contributed to this increased recognition and recording of intellectual disability in English general hospitals. The Equality Act became law in the United Kingdom in 2010, and strengthened the provisions of existing legislation; this Act not only mandates that service providers make reasonable adjustments to ensure that people are not discriminated against (e.g. by nature of a disability) but also states that providers should be proactive in anticipating and making such adjustments [42]. There is also now a legal duty for public sector providers (including the NHS in England) to publish information to demonstrate compliance with the Act, serving as a further incentive for hospital managers to respond to the needs of disadvantaged groups. The English Confidential Inquiry into Premature Deaths of People with Learning Disabilities (CIPOLD), published in 2013, highlighted the premature mortality and preventable deaths that people with an intellectual disability suffer [6] and prompted the establishment of a national learning disability mortality review programme (known as LeDeR) in 2016. Annual LeDeR reports continue to show significant health inequities [43] thereby maintaining attention on the issue and stimulating local and national quality improvement initiatives [44]. A relatively new role of a learning disability liaison nurse has been established in many acute hospitals in England over the past decade. Learning disability liaison nurses undertake a variety of tasks including frontline patient care, educating staff, influencing strategy, and implementing disability-specific recommendations and guidance [45], all of which are likely to raise the profile of people with intellectual disability throughout the hospital [46]. The trend for improved recording may also reflect overall improvements in diagnostic coding in HES data that has been observed over time [47].”

6. The finding relating to married people needs to be treated with caution due to the small number. The reader needs to be reminded of this.

We agree with the reviewer that this finding should be interpreted with caution. We have added to the discussion section to make this clear to readers (p.21, l.418-424):

"People with intellectual disability who were married, cohabiting, or in a civil partnership were also more likely not to have their intellectual disability recorded; it may be that assumptions about the lives of people with intellectual disability contributed to this finding, or that these people had milder intellectual disability which was more easily missed, even though we adjusted for intellectual disability severity. However, this finding should be interpreted with caution owing to the relatively small numbers who were married.”

7. One of the aims of the study was to identify clinical and socio-demographic factors associated with the recording of intellectual disability. I am not sure what clinical factors have been identified; this needs to be clarified.

The clinical factor which we were able to consider in our study was the degree of intellectual disability and we found that those with more severe intellectual disability were more likely to have the intellectual disability recorded in their hospital records.

8. A much broader discussion is required about whether or not a code for intellectual disability is likely to improve the issues related to poor quality care that are listed in the Introduction (lack of knowledge and understanding amongst health professionals, diagnostic overshadowing, institutional discrimination, inadequate communication and failure to acknowledge carers' expertise). There is possibly an argument to be heard that suggests that labelling a person with intellectual disabilities in this way, of itself leads to prejudiced or discriminatory care.

We agree with the reviewer that recognition of intellectual disability does not in itself guarantee suitably-adapted or optimum care, and we have added to the discussion. We also recognise that labelling the disability has potential to lead to stigma and labelling the person’s additional needs or adjustments required is an alternative approach. We have added to the discussion (p.24, l.491-513):

“Recognition of intellectual disability should by law result in suitably adapted care in England, but does not necessarily guarantee a tailored care response, or indeed, result in better outcomes. It is important that awareness of intellectual disability is supplemented by all hospital staff having a broad understanding of the range of health and communication needs that people with intellectual disability may have and being familiar with general approaches to care. Access to specialists should then be available to support clinical teams to with more specific assessments and advice; this might include input from a learning disability liaison nurse or in-reach from a community learning disability team. Furthermore, there needs to be flexibility within the hospital ecosystem to enable truly individualised care, including extra time for interventions and allowing family members or carers to remain with the person where this may ordinarily not be permitted. Aside from these practical interventions, the presence of institutional discrimination against people with intellectual disability [16] demands a cultural shift that may be more difficult to effect and measure but it is hoped that a forthcoming programme of mandatory training in autism and learning disability for all National Health Service staff [55] will challenge misconceptions and contribute to improvements in care.

It is possible that ‘labelling’ the intellectual disability may lead to stigma. A recent review of visual identifiers in the care of people with dementia highlighted potential ethical issues with flagging people with dementia in hospitals, but found that ethical and legal dilemmas could be overcome [56]. Alternatively, it would be possible to flag the domain(s) in which a person requires additional support, thereby labelling the adjustments rather than the disorder. This method could be applied to people with other deficits, such as those arising from dementia or sensory impairments, however, this would negate the ability to identify intellectual disability for legal compliance, research and service planning purposes.”

9. I would also have liked more consideration to be given as to whether coding intellectual disabilities is, in fact, more appropriate than coding the reasonable adjustments required by an individual. People with intellectual disabilities can have very different needs. Although there may be a 'menu' of reasonable adjustments that may be appropriate, what is important is the individual tailoring of these to the needs of an individual. A reflection on whether this is a better way forward than the rather blunt coding of 'intellectual disabilities' would be helpful.

The reviewer also raises an important point about the heterogeneity of the population with intellectual disability and the need for tailored support. Our response to this is covered in the previous response.

10. Finally, an area of confusion for me is that in the section about the study limitations, it is noted that 'This study looks only at recording of intellectual disability in hospital records, using the patient discharge summary as the source of HES data'. This seems to contradict what is written in the Methodology section where it is stated that: 'We used HES inpatient admission data which include diagnoses identified during the hospital contact...' This requires clarifying.

We thank the reviewer for highlighting the potential confusion here. Diagnoses made or recognised at any time during the hospital admission should be added to the discharge summary via electronic records and the discharge summary is then used as the primary source of HES data.

We have amended the method and limitations sections slightly to better reflect this (p9, l.153-155 and p.21, l.461-462):

“We used HES in-patient admission data which include diagnoses identified during the hospital contact which have been added to the in-patient discharge summary…”

“This study looks only at recording of intellectual disability in hospital records, using the patient discharge summary as the primary source of HES data.”

**Reviewer #2:** Alex McConnachie, Statistical Review

Sheehan et al looks at the recording of intellectual disability in routine data for hospital admissions of people with ID in a large mental health trust in England between 2006 and 2019. This review considers the use of statistics in the paper.

Generally, these are quite well done. Outcomes are defined at the admission level and patient level, and are reported for all admissions and non-elective admissions. A strict coding definition and a wider definition are used. Logistic regression is used to assess the factors associated with poorer recording (at the patient level). Multiple imputation is also done, to assess the robustness of the main results. The interpretation of the statistical results are reasonable.

We thank the reviewer for their positive comments and suggestions.

My main worry with the analysis is whether the data are suitable to answer the question. For someone with ID, is it necessarily the case that ID should be recorded in HES data? Are the diagnoses recorded in HES inpatient stay data more to do with the clinical reason for the admission? If the admission has nothing to do with the fact that the patient has ID, is there any reason for ID to be recorded in the HES data? The authors question whether the low level of recording is to do with administrative error, though my point is whether this is an "error" at all. It may be that a patient's ID is appropriately recognised during their admission, but unless there is some connection, this is not recorded as being a reason for their admission, or is only recorded occasionally.

That said, the results are still of interest. They show that HES data are not a reliable source of data about patients' ID status. They raise the question as to whether patients' ID status is appropriately recognised in secondary care, despite the low level of recording in routine data.

The diagnoses contained in HES data should not only be related to the primary reason for admission or the conditions treated during the hospital stay. Up to 20 different diagnoses can be added for each patient record, allowing for multi-morbidity to be recorded. Indeed, there is an incentive for other conditions to be routinely recorded in HES data as a means of ensuring that hospitals are appropriately reimbursed for their activity. Hospital doctors completing discharge summaries should be aware of these requirements and discharge summaries are usually reviewed by senior doctors.

Our assertion is that a diagnosis of intellectual disability is *always* relevant to a person’s healthcare and therefore should be recorded. People with an intellectual disability have significant impairments in global intellect and adaptive functioning, including in expressive and receptive communication, executive functioning. Intellectual disability may impact decision-making capacity and hence the ability to provide informed consent for medical procedures and interventions. People with an intellectual disability, by definition, require additional support, and deficits may be further increased when the person is physically unwell and in a new and unfamiliar environment.

Recording is also of relevance on a wider scale, so that hospitals and commissioners can monitor demand and plan future health services, and it is important that the healthcare needs of people with intellectual disability are also recognised via this route. Our study also informs future researchers whether HES can be a valid source for ascertainment of intellectual disability status.

My remaining comments are minor. Some are simply suggestions for analyses that could be done, and probably reflect my biases as a reviewer. They might be of academic (statistical) interest, without adding much additional insight, or may be considered more complex than is warranted for a general medical audience.

In the logistic regression models, the analysis is at the patient level, and the number of admissions each patient has is adjusted for. How good is this adjustment? Does the log odds of ID being recorded increase in a linear way with the number of admissions?

The skewed distribution of the study participants' number of admissions led us to categorise this variable as we anticipated that it would violate logistic regression assumptions. We therefore used this categorised variable (as shown in table 1, 1; 2-10; >10)) in our regression models. We realise that this was previously not clear and so have clarified this in the methods section where we describe the analysis (p.12, l.229-231):

“Univariate regression was conducted for each variable followed by multivariable analysis adjusted for each co-variate and number of hospital admissions in the study period, categorised as 1; 2-10; >10”

Note that the number of admissions someone has is not a baseline characteristic - it is based on what happens to the patient after the baseline, which always has the potential to lead to bias. E.g., those people who enter the study cohort later on in the study period will have less time to accrue admissions, and therefore fewer opportunities for ID to be recorded, though the authors present analyses that suggest an increase in recording (through the F81.9 code) over time, so it is not clear how these competing factors will affect the patient-level recording of ID.

We agree with the reviewer’s point and have added this to our limitations (p.22, l.461-464):

“Our analysis of factors associated with patient-level recording of intellectual disability is potentially affected by those who enter the study having less time to accrue admissions and recording accuracy changed over time. The consistency of findings in the sensitivity analysis helps to increase confidence in these results.”

Was an admission-level analysis considered? This would avoid the problem noted above, but would have to account for clustering at the patient level. It could, however, assess whether recording is more likely during a non-elective admission, and whether there is a correlation over time in ID recording - i.e., once ID has been recorded during one admission, is it more likely to be recorded in subsequent admissions?

Thank you for this suggestion – we have added a sensitivity analysis examining factors associated with diagnostic recording using mixed effects logistic regression which we describe below in response to comment 4 from reviewer 4. The findings from this were largely in keeping with our primary analysis.

Analysis of the raw data in this and our previous studies using this data source (Mansour 2020, Sommerlad 2018) indicates that recording is more likely in non-elective rather than elective admissions, as these are usually brief and recurrent (e.g. day-case admission for wound dressing or renal dialysis), and that recording is more likely with more admissions. For this reason we adjusted our primary analyses for the number of admissions.

The authors suggest that it is not possible to measure quality of care. However, an analysis could be done looking at the time to the second (non-elective) hospital admission after the first. Whether or not ID was recorded during the first admission could be assessed as a covariate. Are patients whose ID status was recorded during their first admission (and therefore presumably appropriately recognised during that admission), at higher or lower risk of subsequent admissions? Such an analysis could even be done looking at the times from each admission to the next, though this would need to account for patient-level clustering again, and could become quite complex.

The reviewer makes an interesting point. We have considered this within the research team and, on balance, feel that the time between admissions is not a valid proxy for quality of care received during an in-patient admission. People with intellectual disability may have complex conditions that necessitate regular admissions, irrespective of quality of care, and the frequency of admissions is likely to be related to many other factors, including the quality of community health and social care.

In addition, a significant proportion of the sample only had one admission in the study period, and therefore an analysis of readmission data would be limited by smaller numbers.

The discussion notes that a quarter of patients could have a genetic cause for ID, which could be recorded with different ICD codes. This seems like quite a large proportion. Would it be feasible to identify these patients (N ~ 600) and their genetic conditions, to see if these are recorded in the HES data?

We thank the reviewer for this suggestion. We have added to the analysis by including an analysis using a list of ICD-10 codes for specific conditions that are almost always associated with intellectual disability. Including this list resulted in a small increment in the proportion of admissions / people who were recorded. We have described this in more detail in response to comment 4 by reviewer 1.

In Table S1 (and similar), the title says “F70-79 or F81”, but within the table, the term used is “F70-79 and F81”, which is inconsistent.

We are grateful to the reviewer for pointing this out. The title is correct and we have amended the supplementary tables.

**Reviewer #3:**

This paper links two healthcare databases to assess the rate at which intellectual disability has been recorded in hospital records for patients an intellectual disability. This is an extremely well written paper on an important topic. I have only a few minor suggestions for the authors.

We thank the reviewer for their positive comments.

* I would suggest the authors review some of the broader literature on recording of intellectual disability in health records. This can help demonstrate that this is a widespread issue and potentially support speculation on the challenges leading to poor documentation. For example see Lin et al. (2015) Strengths and Limitations of Health and Disability Support Administrative Databases for Population-Based Health Research in Intellectual and Developmental Disabilities

We thank the reviewer for highlighting this paper which we have now included in the discussion (p.23, l.483-489):

“automatic flagging of people with intellectual disability attending hospital could be achieved with better integration of health records between statutory services, underpinned by stringent data sharing protocols and assurances about data confidentiality. A Canadian initiative demonstrated that linkage between health, education, and disability social care databases can capture a greater proportion and more varied group of people with intellectual disability and improve their visibility within statutory services, though the authors note that time and other resource constraints must be overcome as potential barriers to effective data linkage [54].”

* I was surprised to see the much higher use of the F81.9 code. Do the authors have any theories on why this was used at such higher rates and whether there are any clinical consequences of using this code?

Indeed, this was also a finding that we were not expecting. The F81.9 code is a residual category for ill-defined learning disorders or difficulties that do not fit elsewhere. Use of the code may result from confusion around the meaning of the code; abbreviated ICD-10 criteria give synonyms for this code as “learning disability/disorder NOS [not otherwise specified]” which may be misinterpreted. We have added to the discussion (p.18, l.358-362):

“Regular use of this code suggests that some form of cognitive impairment was more frequently recognised during the admission; in practice, this code seems to be used as a proxy for intellectual disability or it may be used when intellectual disability is suspected but not confirmed. Alternatively, it could be used for other unspecified cognitive impairments or borderline intellectual functioning, which does not have a separate category.”

There may be clinical implications of use of the F81.9 code as this does not, strictly speaking, define intellectual disability. Clinicians reviewing patient notes that use this code may not recognise that it has been used to indicate intellectual disability and may not consider the person for reasonable adjustments. Similarly, researchers will need to be aware that applying only F70-F79 codes to HES data is likely to miss a substantial proportion of people with intellectual disability who have been admitted to hospital, and the F81.9 code may also need to be included in future study protocols.

**Reviewer #4:**

The paper entitled "Recording of intellectual disability in general hospitals 2006-2016: cohort study linked datasets" by Sheehan R et al. aimed to 1) investigate the recording of intellectual disability in adults with a confirmed intellectual disability diagnosis who were admitted to general hospitals, 2) analyse changes in the recording of intellectual disability in those admitted to hospital over time and 3) identify clinical and socio-demographic factors associated with intellectual disability being unrecorded in those with the condition.

Overall comment:

In general, this manuscript is well written and well organised to be accessible to non-specialists. However, overall I felt the paper did not elaborate enough on the importance of intellectual disability being recognised in general hospitals. For example, the authors may have benefited from comparing the average length of stay of episodes where intellectual disability is being recognised versus unrecognised. The investigation could highlight and support their claim that recognising intellectual disability is essential for providing additional support and reasonable adjustments in this population.

We thank the reviewer for this idea. As a research team, we have considered the metrics available to us that might be used to extend the work with a measure the quality of care received. Reviewer 2 made a similar suggestion to relate recording to time between admissions (in those who have more than one admission). However, on balance, we felt that there are too many additional factors influencing length of admission that we would not be able to control for to make this a valid proxy for quality of care. For example, we do know that, in the UK, patients with additional disability-related needs often spend longer in hospital than is strictly necessary for medical purposes because of a lack of availability of suitable social care.

I also have some concerns around the methodology used, highlighted below.

The authors might not be aware of one article that examined factors associated with recognising intellectual disability for adults during hospital admission in Australia (Walker AR, Trollor JN, Florio T, Srasuebkul P. Predictors and outcomes of recognition of intellectual disability for adults during hospital admissions: A retrospective data linkage study in NSW, Australia. PLoS One. 2022 Mar 25;17(3):e0266051. doi: 10.1371/journal.pone.0266051. PMID: 35333913; PMCID: PMC8956190.).

We thank the reviewer for highlighting this reference which we have now added to the paper (p.19, l.376-380):

“There is no English benchmark against which the results of this study can be directly compared, although data suggests that low levels of recording of intellectual disability in healthcare records is an international issue. A recent study conducted in New South Wales, Australia, found an overall recognition of intellectual disability in only 23.79% of hospital admissions of adults with the condition, and that recording had reduced over time [37].”

Methods

I have multiple concerns with the method that I believe need to be addressed before publication.

1. Although the authors used strict ICD10 codes to identify intellectual disability, I recommend the codes be broadened to include some conditions like Down Syndrome or other congenital malformations that are linked to intellectual disability.

Thank you for this suggestion. We have now included more comprehensive list of ICD-10 codes for specific disorders associated with intellectual disability. We have described this in more detail in response to comment 4 by reviewer 1.

2. I found that the outcome, which is the failure to recognise intellectual disability, is unintuitive. When the outcome is framed this way (rather than as the positive recognition of intellectual disability), most records have an outcome. In general, logistic regression can perform poorly when a large majority of records have positive outcomes. It also makes it unintuitive for readers to understand the results, as it is predicting the lack of an event. I recommend that the authors change the outcome to intellectual disability being recognised.

We chose to frame our outcome as intellectual disability being *unrecorded,* rather than *recorded* as we considered this as being a marker of potentially adverse care whereby patients may be particularly disadvantaged in a hospital setting. Using this negative outcome highlights the need for future action. However, we would be happy to accept an editorial decision on this matter.

3. I liked having a gold standard and comparing it with another dataset. However, have the authors considered that staff at general hospitals might not know that someone had an intellectual disability before the diagnosis was recorded in the SLaM BRC? Instead of using all HES episodes, authors should use hospital episodes after the formal diagnosis has been recorded in SLaM.

We used the SLaM record to identify the cohort with intellectual disability and are confident that the diagnosis in this dataset is robust. Based on clinical experience, a large proportion of the diagnoses of intellectual disability in the SLaM dataset will not be ‘new’ diagnoses but will have been added to the record as a pre-existing condition when the person first had contact with SLaM adult services. Indeed, intellectual disability is a lifelong condition and it is relatively rare for these services to make a ‘new’ diagnosis of intellectual disability in an adult and as most cases of intellectual disability are diagnosed in childhood, we do not believe that our approach will lead to erroneous results. We have added to the limitations of the paper (p.22, l.455-459):

“It is possible that in some cases, the admission to a general hospital pre-dated the diagnosis of intellectual disability in the SLaM record. However, we consider this number will be low as intellectual disability is a lifelong condition that is most often diagnosed in childhood and the SLaM recording of intellectual disability in an adult is unlikely to be the first point at which the diagnosis is made.”

4. I don't think regular logistic regression is appropriate; authors should consider a random effect model which controls for within-person effect or a generalised estimating equation.

In response to this comment, we ran a mixed effects model and have included this as a sensitivity analysis (supporting information, S3 table and S4 table (with multiple imputation)). We have added to the method with this information (p.12, l.238-244):

“As a sensitivity analysis, we examined factors associated with unrecorded diagnosis during each admission. As these admission-level data have a multilevel data structure whereby many patients had several admissions with correlation between these likely, we used mixed effects logistic regression with a random effect for intercept meaning that the odds radios derived from this analysis reflect the risk of diagnosis being unrecorded during each admission according to each patient characteristic. We then repeated these models using multiple imputation to impute missing socio-demographic or clinical characteristics.”

This analysis supports our primary results that being married and having a less severe degree of intellectual disability is associated with a higher change of the intellectual disability being unrecorded. Other variables were also significant in the adjusted analysis (p.16, l.320-326):

“The results of the sensitivity analysis using a mixed effects regression model are presented in supporting information (table S4 and S5). In common with the logistic regression, the odds of intellectual disability being unrecorded during each hospital admission were lower in those with a greater degree of intellectual disability and higher in those who were married. Being younger and being of Asian ethnicity were independently associated with lower odds of intellectual disability being unrecorded and a higher deprivation index was associated with higher odds of intellectual disability being unrecorded.”

5. The use of the term 'sensitivity' is an interesting choice here. For statisticians, the term 'sensitivity' refers to a given test's ability to correctly designate an individual with a disease (or other binary outcomes) as positive. In this context, I think what the authors presented is the proportion of episodes or people that are correctly identified as having an intellectual disability.

We thank the reviewer for this comment. We accept that we are not discussing the psychometrics of a screening instrument, but we are still considering a gold standard (i.e. the SLaM BRC diagnosis) and the proportion of people positive on the gold standard who are correctly identified as such by a procedure under evaluation (i.e. discharge diagnoses recorded in HES). In analogous studies, we have used the term sensitivity for this purpose (Sommerlad et al, 2018; Mansour et al, 2020), and so we have kept the term for this study.

To avoid any confusion, we have defined our use of the term sensitivity, using the reviewer’s definition, in the method (p.10, l.198-201):

"We calculated the proportion of people or episodes that are correctly identified as having an intellectual disability in general hospital records (HES) after the first recorded diagnosis in the BRC Case Register, which we will hereafter refer to as 'sensitivity'.”

**Reviewer #5:**

This linked data study utilised information from a large secondary mental healthcare database to identify those with intellectual disability and linked these individuals to general hospital records, to investigate how well intellectual disability had been recorded in medical files. I assume therefore that the cohort will be restricted to those individuals with intellectual disability and co-morbid mental health conditions? This may need to be clarified. The study aimed to look at trends over time and factors associated with the non-recording of intellectual disability.

We identified the cohort as those who had had contact with a National Health Service mental health provider and had been diagnosed with intellectual disability. However, not all will have had co-occurring mental illness, as some referred to the service will not receive an additional diagnosis and may be referred for behavioural issues or for other reasons.

We have added to the limitations to comment on the representativeness of the cohort studied (p.22, l.443-448):

“We used diagnosis of intellectual disability made by a secondary mental health care service (which includes specialist intellectual disability teams) as the gold-standard meaning that only individuals with intellectual disability living in the catchment area who have accessed secondary mental health and intellectual disability services have been included; those accessing specialist services are more likely to have additional complex needs and may not be representative of the total intellectual disability population”

Introduction

The introduction has provided evidence of the high morbidity and earlier mortality experienced by individuals with intellectual disability. They also experience a higher number of hospitalisations than those without intellectual disability as has been evidenced across multiple countries. The authors state little information exists on how well intellectual disability is recognised and recorded in general hospital settings and may not be aware of an Australian study which has also investigated how well individuals with intellectual disability were coded as such in hospital records. This study showed only about 15% received an intellectual disability code although it used a younger cohort. (Ref Bourke J, Wong K, Leonard H. Validation of intellectual disability coding through hospital morbidity records using an intellectual disability population-based database in Western Australia. BMJ open. 2018 Jan 1;8(1):e019113.)

We thank the reviewer for highlighting this reference which we have now added to the paper (p.19, l.376-382):

“There is no English benchmark against which the results of this study can be directly compared, although data suggests that low levels of recording of intellectual disability in healthcare records is an international issue. A recent study conducted in New South Wales, Australia, found an overall recognition of intellectual disability in only 23.79% of hospital admissions of adults with the condition, and that recording had reduced over time [37]. A further Australian study that investigated the recording of intellectual disability in children showed that hospital data identified only 14% those with intellectual disability who had been admitted [38].”

Methods

Methods seem appropriate and well-explained. The authors used only ICD codes specific to intellectual disability (F70-79) to identify ID and did not include codes for conditions that are consistent with intellectual disability such as Down syndrome, Fragile X or Prader-Willi. Although some conditions may not be 100% associated with ID this may have explained some of the un-coded ID- particularly for those with Down syndrome. Coding practices may not see the need for an intellectual disability code in those with Down syndrome? I see that this issue has been addressed in the discussion.

The reviewer makes an important point that other reviewers also mentioned. We have now included an additional list of ICD-10 codes that signify specific disorders that are almost always associated with intellectual disability. We have described this in more detail in response to comment 4 by reviewer 1.

Results

Table 2 would probably be clearer if the row labelled 'ICD_10 Codes' was simply included in the row above it and labelled 'ICD-10 Descriptor and codes'.

We thank the reviewer for this suggestion. The table has been amended.

The increase in % identified through ICD codes when F81.9 is included (Table 2) possibly indicates the large proportion of individuals who may have borderline intellectual disability and there is not the confidence to assign a specific level of ID using the F70-79 codes, even though they do include an unspecified level, F79. The time trend (Figure 1) is also interesting to see this code obviously being used more frequently over time.

We have added to the discussion (p.18, l.358-362):

“Regular use of this code suggests that some form of cognitive impairment was more frequently recognised during the admission; in practice, this code seems to be used as a proxy for intellectual disability or it may be used when intellectual disability is suspected but not confirmed. Alternatively, it could be used for other unspecified cognitive impairments or borderline intellectual functioning, which does not have a separate category.”

Discussion

The discussion makes many good points justifying the need for correct coding of intellectual disability in the hospital setting, particularly to aid in communication during treatment and discharge. It is also important to have this information recorded for use in resource planning.

The study is clearly written but does have some limitations in being limited to those individuals in the catchment area of the mental health services. However the results are very useful and an important point made is the limitation in using only hospital coding to identify a cohort of individuals with intellectual disability for any research purpose.

We thank the reviewer for their supportive comments.

Minor typos

Page7, line 130: …diagnosis of intellectual disability (and) was taken as either a record of an ICD-10 code

Thank you. This has been amended.

Page10, line 203: The sample comprised 2,477 adults with intellectual disability who were admitted (to) a general hospital

Thank you. This has been amended.

Add a sentence or two to highlight the most recent few years data.

We have now highlighted the most recent data and incorporated this into a more comprehensive discussion about reasons for changes in rates of recording over time (p.20, l.389-391):

“Our data indicate that recording of intellectual disability has improved over time, if the related (but technically incorrect) code of learning difficulty (ICD-10 F81.9) is included. For example, intellectual disability was recorded in over half of new emergency admissions occurring in 2019.”
