# Supplementary material for: Recording of intellectual disability in general hospitals in England 2006–2019: Cohort study using linked datasets
Source: PLoS Med. 2023 Mar 20;20(3):e1004117. doi: 10.1371/journal.pmed.1004117 (PMC10069786; doi:10.1371/journal.pmed.1004117)
Supplement: Attachment — Submitted filename: Recording of intellectual disability - R2 - author response.docx [file pmed.1004117.s009.docx]

**Recording of intellectual disability in general hospitals in England 2006-2019: cohort study using linked datasets (PMEDICINE-D-22-03204R2)**

**Author response to reviewer comments**

We thank the editor and reviewers for their comments. Comments in black text are followed by author response in red text. We have included additions to the text and given the page and line numbers corresponding to the clean (unmarked) version of the revised manuscript.

**Requests from the editors:**

Abstract Methods and Findings:

* Please ensure that all numbers presented in the abstract are present and identical to numbers presented in the main manuscript text.

We have confirmed the figures presented in the abstract.

* Did your study have a prospective protocol or analysis plan? Please state this (either way) early in the Methods section.

a) If a prospective analysis plan (from your funding proposal, IRB or other ethics committee submission, study protocol, or other planning document written before analyzing the data) was used in designing the study, please include the relevant prospectively written document with your revised manuscript as a Supporting Information file to be published alongside your study, and cite it in the Methods section. A legend for this file should be included at the end of your manuscript.

b) If no such document exists, please make sure that the Methods section transparently describes when analyses were planned, and when/why any data-driven changes to analyses took place.

c) In either case, changes in the analysis-- including those made in response to peer review comments-- should be identified as such in the Methods section of the paper, with rationale.

We have now included our study protocol as supplementary information (S1 text) and noted this in the manuscript (p.8, l.135-137). This document was written before this work was undertaken but was not prospectively published on an externally-kept register.

“The study protocol document, written before the data were extracted and any analysis was performed, is included as a supplementary file (S1 text).”

We have added a new section to the methods detailing changes from the original protocol (p.13, l.256-272):

“*Changes from originally-planned work*

We had planned to also investigate recording of autism as part of this work but restricted our analysis to intellectual disability to make for a more focused paper and coherent narrative. Some changes were made to the protocol after further discussion within the research team but before data extraction or analysis. We extended the date of patient identification to 2019 to permit a larger sample size and so that time trends would become more apparent. We had planned to only include data from emergency admissions but chose to also include elective admission data and to report this separately in the analysis in order to get further insights into rates of recording. We chose not to include Health of the Nation Outcome Scales (HoNOS) data (a clinician-rated measure of psychiatric symptoms and social functioning) owing to high amounts of missing data and because the scale is not designed to capture the full range of potential impairments relevant to people with intellectual disability. We had planned to analyse whether recording of intellectual disability was associated with time to admission. However after further consideration, we felt that time between admissions is not a valid proxy for quality of care received during an in-patient admission, as people with intellectual disability may have complex conditions that necessitate regular admissions, irrespective of quality of care, and the frequency of admissions is likely to be related to many other factors, including the quality of community health and social care.

After the data extraction, we noted the high rates of use of the F81.9 diagnostic code; this was not expected but was felt to be relevant to understanding coding in a clinical context, and therefore these codes were added to subsequent analyses while also presenting findings without this code.

In response to comments of peer-reviewers, we undertook additional analyses which are presented in the final version of the paper. At this stage, we updated the analysis to include a list of ICD-10 codes associated with specific causes of intellectual disability. We also included the sensitivity analysis following comments of reviewers, examining factors associated with diagnostic recording using mixed effects logistic regression.”

**Comments from the reviewers:**

**Reviewer #1:**

Thank you to the authors for their consideration of the reviewers comments and for the clear and transparent way in which they have made changes to this paper in line with the comments. In my view, the paper is now ready for publication.

**Reviewer #2:** Alex McConnachie, Statistical Review

I thank the authors for their consideration of my original comments.

Knowing that the number of admissions is included in the models as a categorical variable is useful, but it would be of interest to see the odds ratios associated with this factor in the adjusted regression models. However, I note that this adjustment is included in the adjusted mixed effects model at the admission level. I am not sure that this makes sense, since the unit of analysis is the admission; why should the total number of admissions be associated with recording within any one admission?

We have added the odds ratios associated the number of admissions in the adjusted and unadjusted analysis in table 3 (page 18-19). The unadjusted odds ratios (for unrecorded diagnosis) are: 1 admission, reference; 2-10 admissions OR 0.38 (95%CI 0.24,0.56, p<0.001); >10 admissions OR 0.19 (95%CI 0.12,0.30, p<0.001). Corresponding odds ratios in the adjusted analysis are: 1 admission, reference; 2-10 admissions OR 0.37 (95%CI 0.20,0.70, p<0.002); >10 admissions OR 0.16 (95%CI 0.09,0.31, p<0.001).

We thank the reviewer for noting this error. We have confirmed that number of admissions was not included in the adjusted admission-level analysis (supplementary tables S4 and S5). The results in the table are therefore correct, but the footer is wrong, and this has now been changed.

It is good to see the admission-level mixed effects model results. These raise a few questions. The age association is reversed - in the main analysis, older age is associated with an increased odds of non-recording, but in the mixed effects analysis, it is associated with a reduced odds of non-recording. This needs to be explained. Also, in the mixed model, I assume that the age variable represents age at the time of the admission, not at baseline?

Age in the admission-level model is age at the time of first hospital admission. We did not adjust for age at each admission as we judged that using this as a covariate would, in a longitudinal analysis largely indicate the effect on recording of individuals being older at each successive admission, and the elevated recording rate at successive admissions would most likely reflect that the hospital record already includes the intellectual disability diagnosis, rather than being a true effect of age.

The following has been added (p.22-23, l.453-465):

“Other factors investigated were not associated with recording in hospital records in the patient-level analysis. However, in the sensitivity (admission-level) analysis, increasing age at first admission and Asian ethnicity were associated with increased recording of intellectual disability; these positive findings may reflect the greater statistical power from this analysis of over 27,000 hospital admissions. The relationship with age may also be due to there being more sources of information available to confirm a diagnosis as someone ages (e.g. social care records). The association with ethnicity suggested in this analysis warrants further investigation, but is also based on small numbers and was no longer significant in the imputed analysis. Higher deprivation levels were associated with decreased recording of intellectual disability in the sensitivity analysis; the lack of a consistent association with socio-economic status may reflect the IMD not being a sufficiently robust measure of deprivation in people with intellectual disability, as many live in group settings which do not reflect the socio-economic status of themselves or their families [48].”

The discussion, around lines 424-428, says that factors other than degree of intellectual disability and marital status were not associated with recording, but in the mixed model, both ethnicity and IMD decile showed evidence of associations. I think the differences between the patient-level and admission-level regression analyses need to be discussed more.

Please see response to previous comment and lines 453-465 in the revised manuscript.

There is a statement around line 430 that recording in elective and non-elective admissions was similar, though I do not recall seeing this tested anywhere. Data are reported for all admissions, and for non-elective admissions, but not for elective admissions, specifically. From the data presented, it appears that recording during non-elective admissions is lower than during elective admissions, if using the strict coding definition, but is higher when including the F81.9 code. The mixed model would be the ideal place to assess this - elective or non-elective could easily be added as a covariate.

We thank the reviewer for suggesting this. We have re-run the mixed models including elective or emergency (non-elective) admission as a covariate (supplementary tables S4 and S5).

An extra sentence has been added to the results section (p.18 l.355-356):

“Unrecorded intellectual disability was almost twice as likely in elective compared with emergency admissions.”

This has also been reflected in an addition to the discussion (p.23, l.467-471):

“Recording of intellectual disability was better in emergency compared with elective admissions. This is consistent with other studies investigating the recording of dementia and severe mental illness [40, 41] and may be due to a more comprehensive medical clerking being undertaken in the emergency department than in planned admissions, or differences in the duration of admissions, giving greater opportunity for the condition to be recognised (a large proportion of elective admissions are for short procedures e.g. dialysis).”

Whilst I am not insisting the analysis be done, I think the response to the suggestion of a time to event analysis did miss the point a little. I specifically suggested that the time to non-elective admission be analysed, so the fact that people with ID require regular admissions is perhaps less relevant. Also, with a time to event (i.e. survival) analysis, not having the event of interest is not a barrier to including people in the analysis.

We thank the reviewer for clarifying this point.

**Reviewer #4:**

The authors did a great job responding to all queries. I do not have any more comments.
